# Ascorbate peroxidase modulation confirms key role in *Leishmania infantum* oxidative defence

**DOI:** 10.1186/s13071-024-06562-5

**Published:** 2024-11-18

**Authors:** Isabella Fernandes Martins Santos, Douglas de Souza Moreira, Karla Ferreira Costa, Juliana Martins Ribeiro, Silvane Maria Fonseca Murta, Ana Maria Murta Santi

**Affiliations:** Grupo de Genômica funcional de Parasitos (GFP), Instituto René Rachou IRR, Fundação Oswaldo Cruz – FIOCRUZ/Minas, Avenida Augusto de Lima 1715, Belo Horizonte, MG 30190-002 Brazil

**Keywords:** *Leishmania infantum*, Ascorbate peroxidase, CRISPR/Cas9, Antioxidant defence, Drug resistance

## Abstract

**Background:**

Ascorbate peroxidase (APX) has emerged as a promising target for chemotherapy because of its absence in humans and crucial role in the antioxidant defence of trypanosomatids. APXs, which are class I haeme-containing enzymes, reduces hydrogen peroxide using ascorbate to produce water and monodehydroascorbate, thereby preventing cell damage caused by H_2_O_2_.

**Methods:**

We aimed to create an APX-knockout *L*. *infantum* line using CRISPR/Cas9. Despite unsuccessful attempts at full knockouts, we achieved deletion of chromosomal copies post-APX episomal insertion, yielding LiΔchrAPX::LbAPX parasites. We performed phenotypic characterization to assess the impact of these genetic modifications, which included the determination of APX transcript expression levels using quantitative PCR, drug sensitivity, infectivity, and parasite survival in macrophages.

**Results:**

Quantitative polymerase chain reaction (PCR) analysis revealed a 10- to 13-fold reduction in APX transcript expression in LiΔchrAPX::LbAPX compared with wild-type (LiWT) and APX-overexpressing (Li::Cas9::LbAPX) parasites, respectively. The episomes in those knockdown parasites remained stable even after 20 drug-free passages in vitro. Li::Cas9::LbAPX parasites showed increased resistance to trivalent antimony (Sb^III^) and isoniazid, reduced tolerance to H_2_O_2_, and unchanged macrophage infectivity compared with LiWT. In contrast, LiΔchrAPX::LbAPX parasites were more sensitive to Sb^III^ and isoniazid, exhibited greater susceptibility to H_2_O_2_-induced oxidative stress, and 72 h post-infection, showed fewer infected macrophages and intracellular amastigotes compared with LiWT parasites.

**Conclusions:**

Our findings hint at the indispensability of APX in *L*. *infantum* and raise the possibility of its potential as a therapeutic target for leishmaniasis.

**Graphical Abstract:**

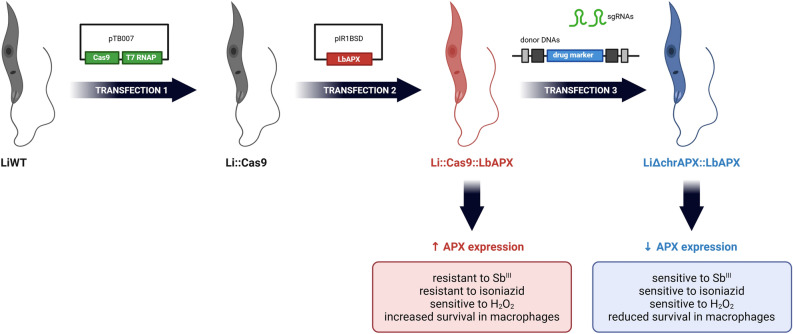

**Supplementary Information:**

The online version contains supplementary material available at 10.1186/s13071-024-06562-5.

## Background

Leishmaniases constitute a set of severe diseases that mostly affect vulnerable populations. They are distributed worldwide and are caused by protozoan parasites of the genus *Leishmania*. Currently, approximately 12 million individuals are infected with this protozoan and over 350 million are at risk of infection [1]. These diseases can be classified as cutaneous leishmaniasis (CL), which is characterised by skin and/or mucosal lesions, and visceral leishmaniasis (VL), in which parasites exhibit tropism for internal organs, such as the liver and spleen, and can be fatal if left untreated [1, 2]. One million cases of CL and more than 30,000 cases of VL are reported annually worldwide [1]. *L*. *infantum*, the focus of this study, is widely distributed and responsible for causing VL.

Only a few drugs are available for the treatment of leishmaniasis, including pentavalent antimonials (e.g. sodium stibogluconate and meglumine antimoniate), amphotericin B and its formulations, miltefosine, paromomycin sulphate and pentamidine isethionate [3]. Chemotherapy for leishmaniasis presents significant challenges owing to high drug toxicity, long treatment protocols, and the occurrence of drug-resistant parasite strains. Therapeutic failure is also associated with the patient’s nutritional status, age, sex and immunity [4]. Therefore, there is an urgent need to identify novel molecular targets for developing drugs against leishmaniasis.

In this context, ascorbate peroxidase (APX) is a promising molecular target for chemotherapy because it is absent in humans and plays an important role in antioxidant defence against trypanosomatids. APXs are class I haeme-containing enzymes that reduces hydrogen peroxide using ascorbate to produce water and monodehydroascorbate in photosynthetic microorganisms, plants and trypanosomatids, such as *Leishmania* spp. [5, 6]. As *Leishmania* lacks the activity of enzymes, such as catalase, selenium-dependent glutathione peroxidase, glutathione reductase and thioredoxin reductase, it relies on the tryparedoxin pathway to survive oxidative bursts during phagocytosis [7]. Studies have shown that the APX enzyme is important for *L*. *major* and *L*. *amazonensis* survival and differentiation within macrophages [6, 8, 9], as well as for protecting *L*. *braziliensis* from the effects of trivalent antimony and increasing its tolerance to oxidative stress [10].

Considering the importance of APX for the parasite, in this study, we investigated the impact of APX overexpression and downregulation in *L*. *infantum* on the parasite growth, susceptibility to anti-leishmanial drugs, tolerance to oxidative stress and the infectivity in THP-1 macrophages.

## Methods

### Parasites and cultivation

Promastigote forms of *L*. *infantum* (MHOM/BR/2002/LPC-RPV) were cultured at 27 °C in M-199 medium (GIBCO^®^), supplemented with 40 mM of HEPES (pH 7.4), 5 μg/mL of hemin, 2 μg/mL of biopterin, 1 μg/mL of biotin, 2 mM of L-glutamine, 500 U of penicillin, 50 μg/mL of streptomycin and 10% heat-inactivated foetal bovine serum. Cultures were maintained by weekly subculturing with the inoculation of 1 × 10^6^ parasites for every 5 mL of medium in 25 cm^2^ culture flasks. All experiments were conducted using promastigote forms in the logarithmic growth phase unless otherwise specified.

### Transfections

The first attempt to knockout (KO) the single copy gene APX (LINF_340005600) in *L*. *infantum* using CRISPR/Cas9 was performed as previously described by Beneke et al. [11]. The *L*. *infantum* line carrying the pTB007 plasmid Li::Cas9, which contains hygromycin as a resistance marker and expresses SpCas9 and T7RNAP, was successfully generated by Santi et al. [12]. The Li::Cas9 line was transfected with donor DNAs and templates for guide RNAs using LeishGEdit (http://www.leishgedit.net/) [11]. Plasmids pTNeo v1 and pTPuro v1, conferring resistance to neomycin (NEO) and puromycin (Puro) antibiotics, respectively, were used for PCR amplification of the donors with 30 bp long homology arms by using primers A and B listed in Additional file 1: Table S1. Single-guide RNA (sgRNA) templates for targeting the 5′UTR and 3′UTR were generated by PCR using primers C and E or D and E, respectively (Additional file 1: Table S1).

For the second attempt to generate endogenous APX *L*. *infantum* deficient mutants, the Li::Cas9 line was transfected with the pIR1BSD_LbAPX vector, which contains blasticidin (BSD) as a resistance marker. This vector was constructed by Moreira et al. [10] and carries a 912 bp fragment corresponding to the coding region of the APX gene of *L. braziliensis* (LbrM.20.0150). The pIR1BSD_LbAPX vector was used in a circular form without digestion and the parasitic line was named Li::Cas9::LbAPX. Subsequently, the Li::Cas9::LbAPX parasites were transfected with donor DNAs and sgRNA templates to KO the chromosomal APX generating the LiΔchrAPX::LbAPX parasites. Plasmids pTNeo v1 and pTPuro v1, conferring resistance to neomycin (NEO) and puromycin (Puro) antibiotics, respectively, were used for PCR amplification of the donor DNA. For this reaction primers A and B were used (Additional file 1: Table S1). Single-guide RNA (sgRNA) templates for targeting the 5′UTR and 3′UTR were generated by PCR using primers C and E or D and E, respectively (Additional file 1: Table S1).

All transfections were performed as previously described [13]. The selection of *Leishmania* clones was performed by plating the parasites in semi-solid M-199 medium and adding selective drugs as per mutant resistance markers as follows: 40 µg/mL of G418 (Gibco), 400 µg/mL of hygromycin B (Invitrogen), 10 µg/mL of blasticidin (Gibco) or 30 µg/mL of puromycin (Gibco). Subsequently, selection drugs were used in weekly passages of the cultures; however, all experiments to assess the parasite phenotype were conducted in the absence of selection drugs. The genomic DNA of all parasites was extracted using the DNAzol reagent (Thermo Fisher Scientific), following the manufacturer’s instructions. Deletion assessment was performed by PCR using primers to amplify the APX coding sequence (primers F and G, Additional file 1: Table S1) and evaluate the replacement of APX alleles with resistance marker sequences (primers H to K, Additional file 1: Table S1).

### RT-qPCR

To investigate transcript levels, quantitative reverse-transcription PCR (RT-qPCR) analysis was performed using cDNA from mutants and LiWT parasites. Promastigotes (approximately 1 × 10^8^) were resuspended in 1 mL of TRIzol^™^ Reagent (Invitrogen), and total RNA was extracted using the chloroform method. The RNA was treated with a Turbo DNA-free Kit (Invitrogen) according to the manufacturer’s instructions, and complementary DNA (cDNA) was obtained using Superscript II reverse transcriptase (Invitrogen) following the manufacturer’s instructions. All cDNA samples were diluted to 50 ng/μL and used in the RT-qPCR amplification reaction using Power SYBR Green Master Mix (Applied Biosystems). The specific primers are listed in Additional file 1: Table S1 (primers L to S). Relative quantification of the target genes in the mutants was compared with that in the LiWT parasite background using the constitutive DNA polymerase gene (LINF_160021500) as a normaliser. Amplifications were performed using the QuantStudioTM 12 Flex system (Thermo Fisher Scientific), following the standard cycling of the machine. Data were analysed using the comparative CT method (2-ΔΔCT) [14].

### Growth curve

To determine parasite growth, 1 × 10^6^ promastigotes/mL were inoculated into the M-199 medium (GIBCO^®^) and maintained at 27 °C for daily counting using a Z1 Coulter Particle Counter (Beckman Coulter).

### EC_50_ assays

To assess the parasite’s susceptibility to trivalent antimony (Sb^III^) (Sigma), H_2_O_2_ (Sigma), and isoniazid (Second Pharma), 2 × 10^6^ promastigotes were incubated in 1 mL of M-199 medium (GIBCO^®^) containing different drug concentrations: Sb^III^ was used at concentrations ranging from 25 to 200 µM, hydrogen peroxide (H_2_O_2_) was used from 10 to 200 µM and isoniazid was used from 200 to 2000 µM. The number of parasites grown in the absence or presence of the drugs after 48 h of incubation was determined using a Z1 Coulter Particle Counter (Beckman Coulter). The effective concentration necessary to reduce growth by 50% (EC_50_) was determined from three independent experiments performed in triplicate. The EC_50_ was determined through the non-linear regression–variable slope model, using the ‘log (inhibitor) versus response’ equation.

### Macrophages infection

To assess the infectivity capacity of mutant parasites, THP-1 cells were cultured in complete RPMI-1640 medium (supplemented with 10% foetal bovine serum, 100 U/mL of penicillin and 100 μg/mL of streptomycin) in a 5% CO_2_ incubator at 37 °C. Human monocytic THP-1 cells were differentiated into macrophages by adding 20 ng/mL of phorbol myristate acetate (PMA) (Sigma-Aldrich). After 72 h, the macrophages were infected with stationary-phase promastigotes of *L*. *infantum* (ten parasites/macrophage) for 3 h. Parasites that failed to infect the cells were removed by washing, and the infected macrophages were incubated in RPMI-1640 medium for 72 h. Infectivity was assessed immediately after 3 and 72 h of incubation. Coverslips were stained with rapid panoptic (Laborclin) and photographed. Infection was quantified by counting intracellular amastigotes using ImageJ software.

### Statistical analysis

All experiments were conducted in triplicate and data were analysed using GraphPad Prism 9.0 (Graph Pad Software Inc.). One-way analysis of variance (ANOVA) or two-way ANOVA were used to assess overall differences between groups, followed by the Bonferroni post hoc test to compare the mutants with the LiWT or the Li::Cas9::LbAPX. Statistical significance was set as *p* < 0.05. *p*-Values were reported following the format of Graph Pad Prism 9.0 (Graph Pad Software Inc), where ns (*p* > 0.05), *(*p* ≤ 0.05), **(*p* ≤ 0.01), ***(*p* ≤ 0.001), ****(*p* ≤ 0.0001).

## Results

### Crispr-based APX Knockdown

The first attempt to generate APX-KO *L*. *infantum* mutants was performed using the CRISPR/Cas9 system, following the methodology developed by Beneke et al. [11]. The *Li::Cas9* line was transfected with equimolar amounts of sgRNA and either one donor DNA for NEO or PURO, or with sgRNA and both donor DNAs. However, only the parasites transfected only with the donor DNA for NEO survived in the presence of neomycin. Parasites transfected with the donor DNA for PURO did not survive in the presence of puromycin.

The presence of the NEO cassette and its correct integration into the genome of *L*. *infantum*, replacing APX, was confirmed by PCR in all the mutant clones tested (Additional file 2: Figure S1 a) by using primers H and J. Another PCR was performed using primers F and G to confirm the deletion of APX in these mutant clones; however, a 775 bp fragment corresponding to the APX coding sequence was amplified (Additional file 2: Figure S1 b). This indicated the retention of at least one allele of the gene in the mutant parasites. In addition, RT-qPCR using specific primers for LiAPX (primers L and M) revealed the presence of APX transcript levels in the mutant parasites (Additional file 2: Figure S1 c). This first attempt was unsuccessful because the APX gene was maintained in the genome of the parasites, suggesting that APX is an essential gene for *L*. *infantum*.

A second attempt to obtain endogenous APX *L*. *infantum* null mutants was to express the APX gene in episomal form and then to KO APX chromosomal copies using CRISPR/Cas9. Initially, *L*. *infantum* parasites expressing *Sp*Cas9 constitutively [12] were transfected with the pIR1BSD_LbAPX plasmid containing the APX gene sequence from *L. braziliensis* [10]. Additional file 3: Figure S2 presents a multiple sequence alignment performed using CLUSTAL O (1.2.4), comparing the protein sequences of LINF_340005600 from *L*. *infantum* and LbrM.20.0150 from *L. braziliensis*. Additional file 4: Figure S3 displays the structural models of the proteins predicted by AlphaFold. Panel (a) shows the predicted structure for LINF_340005600, and panel (b) shows the predicted structure for LbrM.20.0150.

**Fig. 1:**
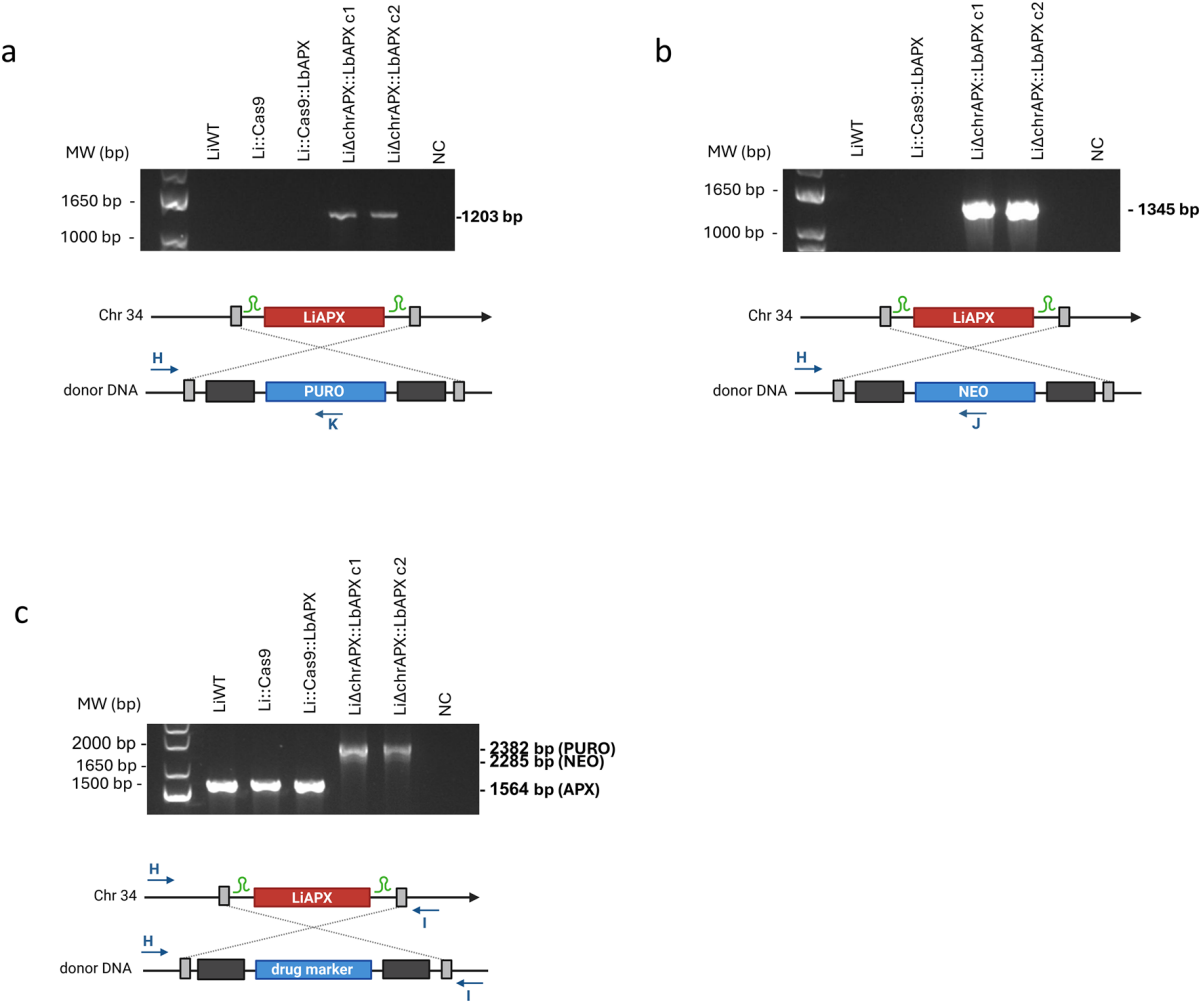
Knockout of APX endogenous gene in *L*. *infantum* expressing *L*. *braziliensis* APX epissomally. First, knockout was assessed by PCR using genomic DNA from wild-type and mutant parasites. The correct integration of the **a** PURO and **b** NEO resistance markers were evaluated by PCR using a primer that anneals in the 5′ UTR region of APX and another primer that anneals in the resistance marker sequence. **c** Integration of donor DNAs using primers that hybridize to the 5′ and 3′ UTR regions of both wild-type and mutant parasites. Please note that the schematic representations of the molecules are not to scale, with sizes and distances adjusted for clarity rather than accuracy. *MW* molecular weight standard, *bp* base pairs, *NC*: negative control, *WT* wild type

In the second knockout attempt, parasites expressing Cas9 and exogenous LbAPX were transfected to knock out the endogenous APX gene. The Li::Cas9::LbAPX clone was transfected with donor DNAs and gRNA templates to generate APX chromosomal null mutants. Then, the LiΔchrAPX::LbAPX mutant clones 1 and 2 were subjected to PCR to verify the integration of the donor DNAs into the alleles containing the APX gene. The correct integration of the PURO and NEO molecular markers was confirmed with specific primers for the 5′UTR region of the APX gene adjacent to the donor DNA (primer H) and the coding sequence of NEO or PURO (primers J or K), generating fragments of 1345 bp and 1203 bp, respectively, in the mutant clones (Fig. 1a, b). In addition, using specific primers for the 5′ and 3′ UTR regions of the APX gene (primers H and I), a amplification of a 1564 bp fragment in the LiWT and Li::Cas9 parasites was observed, whereas both mutant clones contained two fragments each, of 2285 bp and 2382 bp, confirming the correct integration of both donor DNAs into the genome of mutant parasites (Fig. 1c).

**Fig. 2:**
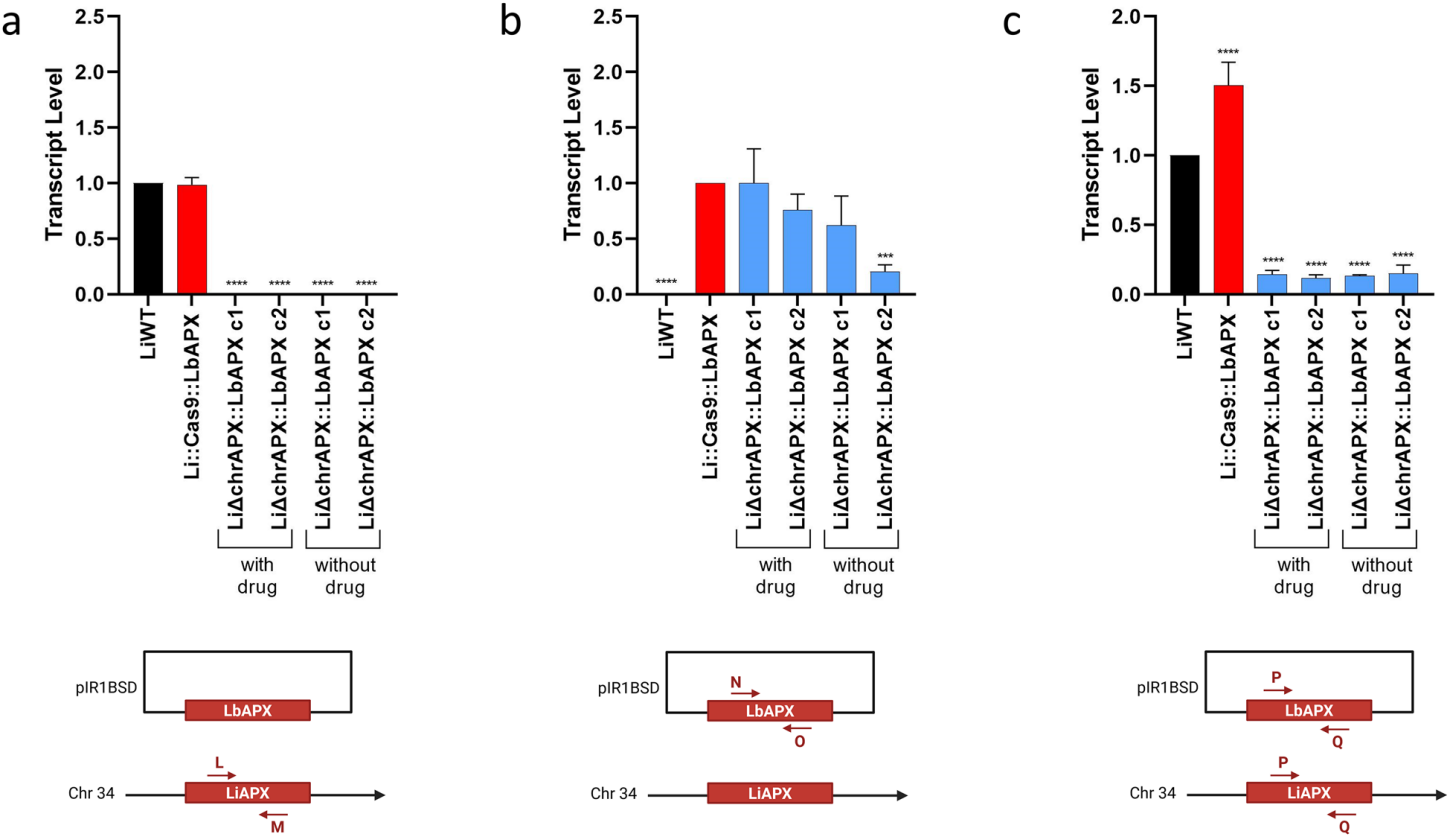
Transcription levels of APX were assessed by RT-qPCR in wild-type parasites and mutants. **a** Transcription levels of LiAPX gene in wild-type and mutant parasites, assessed using a pair of primers that only amplify LiAPX; **b** Transcription levels of the LbAPX gene wild-type and mutant parasites, assessed using a pair of primers that only amplify LbAPX; **c** Transcription levels of both LiAPX and LbAPX in wild-type and mutant parasites, assessed using a pair of primers that recognize both sequences. The transcription level was determined quantitatively to the number of copies of the constitutive DNA polymerase gene by real-time quantitative PCR using the 2-ΔΔCT comparative method. One-way ANOVA with Dunnett post hoc test was used to compare the control parasites and mutants. We used LiWT as the control for **a** and **c** and Li::Cas9::LbAPX as the control for **b**; *p < 0.05, **p < 0.01, ***p < 0.001, and ****p < 0.0001. The sgRNA is depicted in green and indicates the site of the double-strand break within the target sequence. Please note that the schematic representations of the molecules are not to scale, with sizes and distances adjusted for clarity rather than accuracy

RT-qPCR was used to differentiate between chromosomal LiAPX (original APX copies from *L*. *infantum*) and episomal LbAPX (ectopic copies from *L. braziliensis*). Using specific primers for APX in *L*. *infantum* (primers L and M), the APX transcript level was only observed in the LiWT line and Li::Cas9::LbAPX parasites. No transcript level of endogenous APX was detected in the LiΔchrAPX::LbAPX clones (Fig. 2a). In contrast, using specific primers for LbAPX (primers N and O, Additional file 1: Table S1), APX transcript levels were observed in all lines tested, except for the LiWT line, which did not contain the pIR1BSD_LbAPX plasmid (Fig. 2b). This suggests that deletion of endogenous APX in *L*. *infantum* lines was only possible because of the presence of LbAPX in the episomal form.

To verify whether the mutant parasites LiΔchrAPX::LbAPX could lose this plasmid during cultivation without drug pressure, the parasites were maintained by performing weekly passages in a medium without the drug. After 20 passages, the transcript levels of APX were evaluated in these *L*. *infantum* lines using RT-qPCR. Interestingly, using specific primers for APX of *L*. *braziliensis*, APX transcripts were still detected in the LiΔchrAPX::LbAPX parasites even after the 20 passages without the drug (Fig. 2b). Although APX expression in the episomal form decreased during drug-free cultivation of one of the clones, it was not lost. We also evaluated the overall expression of APX using primers that amplify APX fragments common to both species (primers P and Q). The results showed low APX transcript levels in the LiΔchrAPX::LbAPX parasites (Fig. 2c). The difference in the APX transcript levels in the LiΔchrAPX::LbAPX clones was 10.4- and 12.7-fold lower compared with LiWT and Li::Cas9::LbAPX parasites, respectively.

The growth of promastigote forms of LiWT, Li::Cas9::LbAPX, and LiΔchrAPX::LbAPX was monitored for 9 days (216 h). No differences in parasite growth were observed among the lines cultivated in the presence or absence of the drug (Additional file 5: Figure S4).

### APX knockdown impacts parasite sensitivity to Sb^III^, H_2_O_2_, and isoniazid

**Fig. 3:**
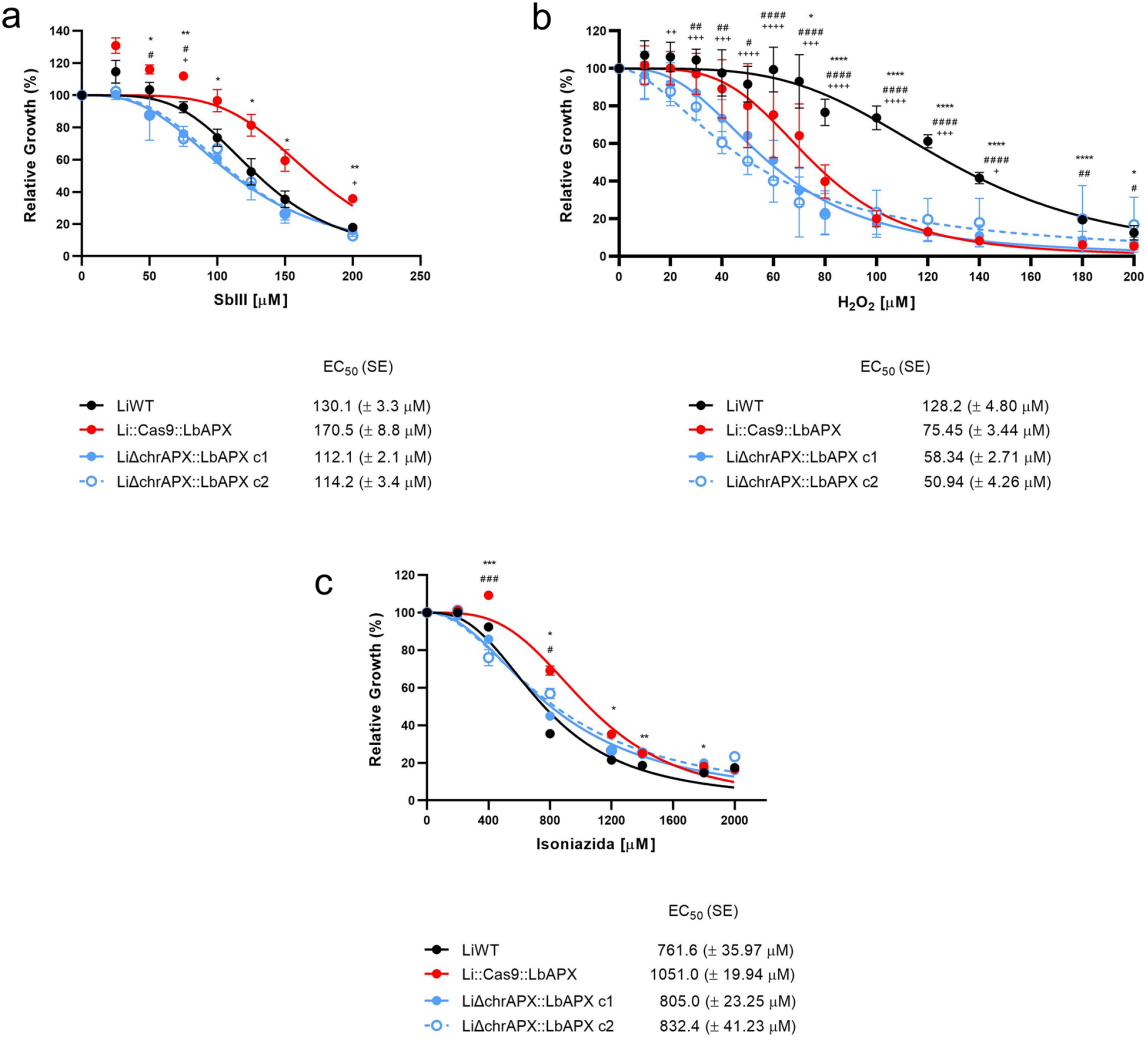
Drug susceptibility. WT and mutant parasites were cultured in the presence of different concentrations of **a** Sb^III^ (25–200 μM),** b** hydrogen peroxide (10–200 μM) and **c** isoniazid (200–2000 μM). Parasite growth was determined after 48 h of incubation with or without the drug. Data plotted in the dose–response curve represent the mean with standard deviations of three independent experiments performed in triplicate. The EC_50_ was determined using a non-linear regression–variable slope model with the ‘log (inhibitor) versus response’ equation in GraphPad Prism. Two-way ANOVA with Dunnett post hoc test was used to compare WT parasites and mutants for each drug concentration**;** *p < 0.05, **p < 0.01, ***p < 0.001, and ****p < 0.0001 for LiWT vs. Li::Cas9::LbAPX; #*p* < 0.05, ##*p* < 0.01, ###*p* < 0.001, and ####*p* < 0.0001 for LiWT versus LiΔchrAPX::LbAPX c1; and + *p* < 0.05, + + *p* < 0.01, + + + *p* < 0.001, and + + + + *p* < 0.0001 for LiWT versus LiΔchrAPX::LbAPX c2

We also investigated whether the low expression of APX in *L*. *infantum* mutant lines altered the susceptibility of parasites to the leishmanicidal drug Sb^III^. The mutant Li::Cas9::LbAPX showed a 1.3-fold higher resistance to Sb^III^ compared with that of the LiWT parasite, with EC_50_ values of 170.5 (± 8.8) and 130.1 (± 3.3) μM, respectively. Clones LiΔchrAPX::LbAPX 1 and 2 were 1.52- and 1.49-fold more susceptible to Sb^III^ compared with Li::Cas9::LbAPX, with EC_50_ values of 112.1 (± 2.1) and 114.2 (± 3.4) μM, respectively (Fig. 3a).

We assessed the tolerance of the mutant parasites to oxidative stress generated by H_2_O_2_. LiWT parasites showed a 1.7-fold higher resistance to H_2_O_2_ compared with that of the mutants Li::Cas9::LbAPX with an EC_50_ value of 128.2 (± 4.8) and 75.45 (± 33.44) μM, respectively. However, parasites deficient in endogenous APX (LiΔchrAPX::LbAPX cl and LiΔchrAPX::LbAPX c2) were 1.3- and 1.5-fold more susceptible to H_2_O_2_ compared with Li::Cas9::LbAPX, with EC_50_ values of 58.34 (± 2.71) and 50.94 (± 4.26) μM, respectively (Fig. 3b).

We also evaluated the susceptibility to isoniazid. Li::Cas9::LbAPX line showed higher susceptibility to isoniazid compared with the LiWT line, with EC_50_ values of 1051(± 19.94) μM and 761.6 (± 35.97) μM, respectively (Fig. 3c). LiΔchrAPX::LbAPX clones 1 and 2 were 1.3-fold more susceptible to isoniazid compared with Li::Cas9::LbAPX, with EC_50_ values of 805 (± 23.25) and 832.4 (± 41.23) μM, respectively (Fig. 3c).

### APX knockdown leads to reduced parasite viability in macrophages

**Fig. 4:**
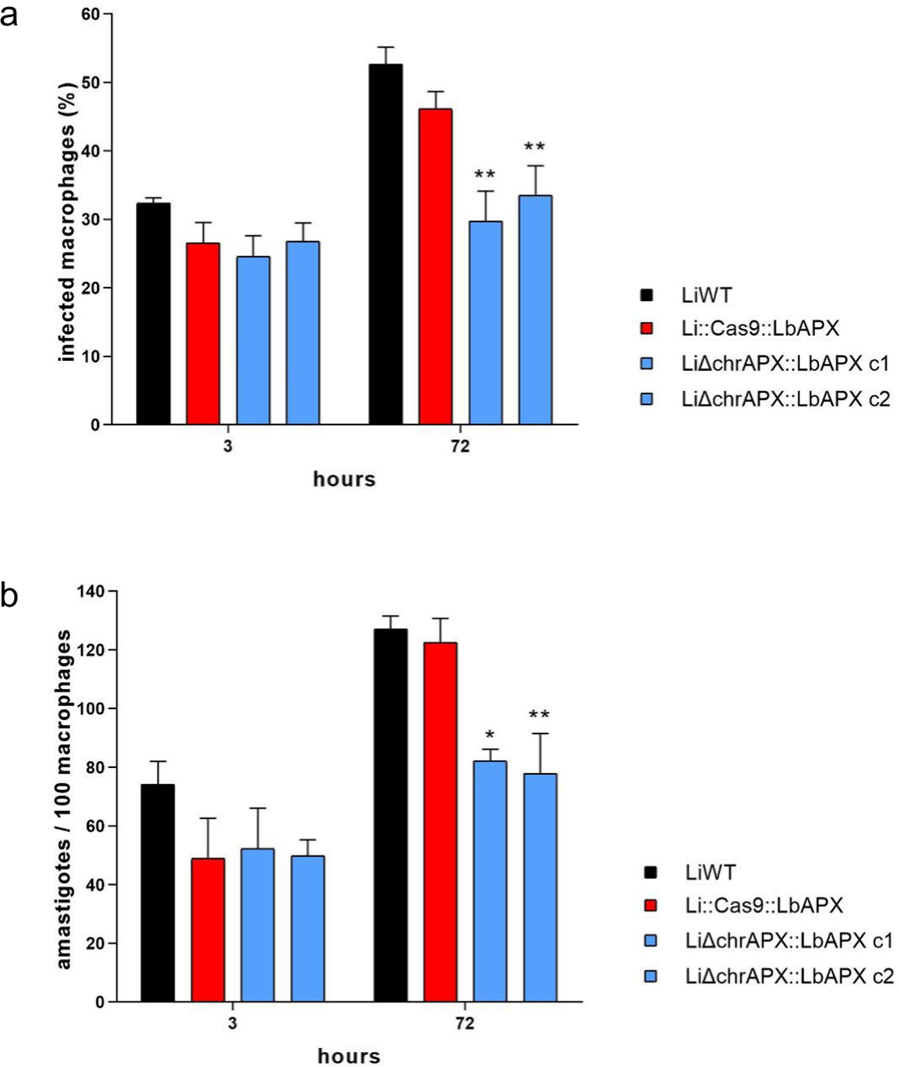
Analysis of the infectivity of mutant parasites in THP-1 macrophages. To evaluate the fitness of the mutant parasites, THP-1 macrophages were infected with LiWT and mutants in a ratio of 1:10 (ten parasites for each macrophage). **a** Percentage of infected macrophages at different incubation periods. **b** Number of intracellular amastigotes per 100 macrophages at different incubation periods. The data are based on the average of three independent experiments performed in triplicate. Two-way ANOVA with Bonferroni post hoc test was used to compare LiWT with each mutant parasite at each time point; *p < 0.05, **p < 0.01, ***p < 0.001 and ****p < 0.0001

We also investigated the effect of low endogenous APX expression *L*. *infantum* on infection profile and intracellular parasite proliferation in THP-1 macrophages. At 3 h after infection, no differences in the percentage of infected macrophages or the number of intracellular amastigotes were observed. However, 72 h after infection, we verified that LiΔchrAPX::LbAPX clones 1 and 2 had a reduced number of infected macrophages and intracellular amastigotes compared with the LiWT line and Li::Cas9::LbAPX (Fig. 4a, b, respectively).

## Discussion

In *Leishmania infantum*, the APX gene typically exists as a single copy within the genome, located on chromosome 34. In this study, we attempted to generate an APX knockout in the *L*. *infantum* line using CRISPR/Cas9 methodology [11]. However, gene was retained, probably because of aneuploidy or gene amplification [15]. The inability to delete APX in *L*. *infantum* suggests that this gene plays an essential role in the parasite. In *Leishmania* parasites, the gene copy number can be altered by adding or removing genes simultaneously or by creating extrachromosomal copies, which are prevalent in *Leishmania* under stress but are also observed in wild-type populations. When attempting to remove important genes, the genetic plasticity of *Leishmania* may cause more gene copies to be generated [16].

Interestingly, the essentiality of APX appears to differ by *Leishmania* species. Pal et al. [8] used conventional allelic replacement to obtain APX-KO *L*. *major* lines. These mutant parasites (LmAPX^−/−^) exhibited an increase in the expression of glutathione peroxidase (nsGPX) and iron superoxide dismutase (FeSOD) enzymes. This result suggests that deletion of the APX gene may lead to upregulation of enzymes involved in the antioxidant defence system of the parasite, as it plays a crucial role in regulating reactive oxygen species (ROS). Xiang et al. [9] attempted to generate APX knockout parasites in *L*. *amazonensis* but could only obtain hemi-knockout parasites for APX (LaAPX^−/+^). These parasites, with a 50% reduction in APX enzyme levels, had impaired infectivity and were unable to induce cutaneous lesions in in vivo models [9]. These findings highlight the importance of APX in *L*. *amazonensis* and are consistent with the results of the present study.

After obtaining clones of *L*. *infantum* with deletion of the endogenous APX and expression of episomal APX (LiΔchrAPX::LbAPX), an attempt was made to remove the p1RIBSD_LbAPX plasmid that expresses APX episomally. The results showed that even after 20 passages (approximately 3 months) of the parasites in the M199 medium without BSD pressure to maintain the episomal plasmid, there was a decrease in the transcript level of the APX gene in *L*. *braziliensis*. This indicated that the number of plasmid copies decreased, remaining at the basal concentration necessary for cell survival. However, no total plasmid loss was observed, suggesting that APX is essential for the survival of *L*. *infantum*. Similar to our results, a previous study attempting to generate null mutants of *L*. *infantum* for the dihydrofolate reductase thymidylate synthase (DHFR-TS) and pteridine reductase (PTR1) genes generated parasites that retained copies of these genes through aneuploidy [17].

*Leishmania* parasites thrive in hostile environments with toxic species and are often exposed to ROS from cellular metabolism, host immune responses and drug metabolism [18–22]. In this threatening scenario, the APX enzyme is part of the parasite antioxidant system, reducing ascorbate and converting H_2_O_2_ into water molecules, aiding in the cellular redox balance [5, 23].

In this study, we obtained mutant clones with decreased expression of APX (LiΔchrAPX::LbAPX) and analysed their phenotypes to understand the role of APX in *L*. *infantum* response to oxidative stress, drug pressure, and infectivity. The decrease in APX transcript levels in *L*. *infantum* did not interfere with parasite growth, whereas the deletion of one allele of APX in *L*. *amazonensis* decreased parasite growth by 50% [9].

Peroxidase-deficient parasites are more susceptible to death upon exposure to H_2_O_2_ [6, 8]. Our results showed that *L*. *infantum* mutant clones with low APX expression (LiΔchrAPX::LbAPX) were more sensitive to H_2_O_2_ than the LiWT parasites. These findings support previous studies showing that APX-KO *L. major* lines are more susceptible to H_2_O_2_ [8]. Studies have demonstrated that *L. major* and *L*. *braziliensis* overexpressing the APX enzyme are more resistant to H_2_O_2_ [10, 24]. However, in this study, we observed that *L*. *infantum* overexpressing the APX enzyme were more susceptible to H_2_O_2_. The balance between SOD and APX activities in the parasites is crucial for determining the steady-state levels of superoxide radicals and hydrogen peroxide. This equilibrium is finely regulated [25]. Thus, we hypothesize that parasites that express high levels of APX may have adapted to a shifted steady state characterized by lower levels of H_2_O_2_ than the WT parasites. Consequently, for these mutant parasites, the sudden introduction of elevated H_2_O_2_ levels may significantly disrupt their steady state compared with WT parasites, despite the higher H_2_O_2_. Therefore, these mutant parasites may exhibit a delayed response to H_2_O_2_. Interestingly, imbalanced oxidant levels can also hinder cell-cycle initiation, thus disrupting proliferation and differentiation [25], which may also have contributed to the lower cell density observed in the APX overexpressing parasites.

APX-deficient *L*. *amazonensis* parasites are unable to sustain their replication in macrophages, whereas APX-overexpressing parasites have increased survival rates both in vitro and in vivo [9]. Results of the present study demonstrate that *L*. *infantum* mutant clones with low APX expression (LiΔchrAPX::LbAPX) had a reduced ability to multiply or survive inside macrophages. This indicates that APX is necessary for parasite survival and that the APX-mediated breakdown of H_2_O_2_ is essential for the intracellular stages of *L*. *amazonensis* and *L*. *infantum* [9, 26–28].

Interestingly, the APX copy number appears to be related to antimony resistance. For instance, parasites resistant to Sb^III^ exhibit intrachromosomal amplification of a subtelomeric locus harbouring APX [29]. Moreover, it was not possible to generate APX-KO parasites in Sb^III^-resistant *L. major* lines [29]. Therefore, we also assessed the susceptibility of *L*. *infantum* mutant clones (LiΔchrAPX::LbAPX) to Sb^III^ and isoniazid. Low expression of APX made parasites more susceptible to Sb^III^, whereas overexpression made them more resistant to this compound, which is consistent with previous studies showing that *L*. *braziliensis* parasites overexpressing APX became eight times more resistant to Sb^III^ [10]. Another study showed that amphotericin B-resistant *L*. *donovani* lines showed increased APX expression compared with susceptible lines [30]. These data support the mechanism of action of Sb^III^; APX is part of the parasite antioxidant system, and a reduction in its expression increases the sensitivity of the parasite owing to disturbances in its redox potential, whereas overexpression provides greater resistance to the toxic effects of the drug. Additionally, these data indicate that APX may play a crucial role in the endogenous detoxification of ROS and is important for the resistance of the parasite to Sb^III^.

Isoniazid is a prodrug derived from isonicotinic acid used to treat tuberculosis [31, 32]. This compound interacts with the APX amino acid sequence of *Leishmania*; its activity was initially tested against *L*. *braziliensis* [10]. Our results showed that the APX-overexpressing *L*. *infantum* clone (Li::Cas9::LbAPX) was more resistant to isoniazid than the LiWT parasite. On the other hand, the mutant clones of *L*. *infantum* with low APX expression (LiΔchrAPX::LbAPX) were more susceptible to isoniazid when compared with the Li::Cas9::LbAPX clone. These findings support the data obtained by Moreira et al. [10], who demonstrated that APX overexpression in *L. braziliensis* rendered the parasites more resistant to isoniazid.

Together, these findings suggest that APX is an essential gene in *L*. *infantum*, making it a viable target for treatment [7, 33]. Remarkably, efforts have been made to identify compounds capable of inhibiting APX [10, 34, 35].

## Conclusions

In this study, we suggested that the APX enzyme might be essential for *L*. *infantum*. An attempt to delete APX using clustered regularly interspaced short palindromic repeats (CRISPR)/Cas9 generated parasites that retained the APX gene. Endogenous deletion of APX was only possible when the parasites exhibited episomal expression of the enzyme. *L*. *infantum* knockout for endogenous APX expressing episomal APX (LiΔchrAPX::LbAPX) showed a tenfold decrease in APX expression and became more sensitive to Sb^III^ and isoniazid. These parasites were also more susceptible to oxidative stress generated by H_2_O_2_ and exhibited reduced survival in macrophages than the LiWT parasites. These results indicate that APX is an essential enzyme for the survival of *L*. *infantum* and reinforces its crucial role in the defence against oxidative stress in these parasites.

## Supplementary Information

Additional file 1: Table S1. List of primers used in this study.

Additional file 2: Figure S1. Attempted knockout of the APX gene in wild-type *L*. *infantum*. The knockout was assessed by PCR using genomic DNA from wild-type and mutant parasites. The correct integration of the a NEO resistance marker was evaluated by PCR using a primer that anneals in the 5' UTR region of APX and another primer that anneals in the resistance marker sequence. b Amplification of the APX coding sequence. c Transcription levels of LiAPX gene in wild-type and mutant parasites, assessed using a pair of primers that amplify LiAPX. The sgRNA is depicted in green and indicates the site of the double-strand break within the target sequence. Please note that the schematic representations of the molecules are not to scale, with sizes and distances adjusted for clarity rather than accuracy. *MW* molecular weight standard, *bp* base pairs, *NC* negative control.

Additional file 3: Figure S2. CLUSTAL O (1.2.4) multiple sequence alignment comparing the protein sequences of LINF_340005600 and LbrM.20.0150.

Additional file 4: Figure S3. The structural model of the protein predicted by AlphaFold, where a) LINF_340005600 and b) LbrM.20.0150. The color-coded regions represent the per-residue confidence scores (pLDDT) predicted by AlphaFold. The pLDDT scores range from 0 to 100, indicating the model’s confidence in the predicted structure of each residue. Some regions with low pLDDT may be unstructured in isolation.

Additional file 5: Figure S4. Growth of LiWT and mutant parasites. Initially, 1×10^6^ parasites per mL were inoculated in M-199 medium. The parasites were cultivated, and the growth was evaluated by daily counting the parasites using the Z1 Coulter Counter. The data presents the average of three independent experiments performed in triplicate, and the growth curves were built using a nonlinear regression model with the “beta growth then decay” equation in GraphPad Prism 9.0.

## Data Availability

No datasets were generated or analysed during the current study.

## Abbreviations

APX: Ascorbate peroxidase
BSD: Blasticidin-S deaminase
HYG: Hygromycin phosphotransferase
H_2_O_2_: Hydrogen peroxide
NEO: Neomycin phosphotransferase
PURO: Puromycin-N-acetyltransferase
Sb^III^: Trivalent antimony
