# Supplementary material for: Ascorbate peroxidase modulation confirms key role in Leishmania infantum oxidative defence
Source: Parasit Vectors. 2024 Nov 18;17:472. doi: 10.1186/s13071-024-06562-5 (PMC11575162; doi:10.1186/s13071-024-06562-5)
Supplement: Supplementary file 1 — Additional file 1: Table S1. List of primers used in this study. [file 13071_2024_6562_MOESM1_ESM.docx]

**Additional file 1: Table S1**. List of primers used in this study

|  | **PRIMER NAME** | | **PRIMER SEQUENCE 5’ → 3’** | **TM (°C)** | **FRAGMENT (bp)** |
| --- | --- | --- | --- | --- | --- |
| Donor DNA | **A** | Upstream FW | GAACTCTGCGAGGAGCACTCCTGCTGACCAgtataatgcagacctgctgc | 60 | pTNEO 1757  pTPURO 1854 |
|  | **B** | Downstream RV | CGCCGTTGACGGAGGCACGTTGTGCAGCTGccaatttgagagacctgtgc |  |  |
| sgRNA | **C** | 5’ sgRNA | gaaattaatacgactcactataggCAAGCACAGCGGAACATGATgttttagagctagaaatagc | 60 | 124 |
|  | **D** | 3’ sgRNA | gaaattaatacgactcactataggTGCTTCTCCTGTAGAGGGCGgttttagagctagaaatagc |  |  |
|  | **E** | G00.rv | aaaagcaccgactcggtgccactttttcaagttgataacggactagccttattttaacttgctatttctagctctaaaac |  |  |
| APX | **F** | LiAPX_FW | ACCTTCGTGAGTGGTGCCAT | 60 | 775 |
|  | **G** | LiAPX_RV | TGAAGCGGTCGTTGTCCTTT |  |  |
| Integration PCR | **H** | 5’UTR LiAPX FW | CAAGCCGAGGTCTCTCTTTAC | 60 | PURO 2382  NEO 2285  APX 1564  PUROmidi 1203  NEOmidi 1345 |
|  | **I** | 3’UTR LiAPX RV | GTGTCAGTCACCCTTCCATTAT |  |  |
|  | **J** | NEO_mid_RV | GCCAACGCTATGTCCTGATA |  |  |
|  | **K** | PURO_mid_RV | CCTCTACTCCTGGAAGAACAAC |  |  |
| qPCR | **L** | qPCRLiAPX_FW | GGTGAGTGCCACATCAAATTC | 62 | 102 |
|  | **M** | qPCRLiAPX_RV | CCAATCCTCATCGAGCAACT |  |  |
|  | **N** | qPCR_LbAPX_FW | TACATGGGTGGTCCGAGTAT | 62 | 113 |
|  | **O** | qPCR_LbAPX_RV | ACGTGATCCTGCGTCTTTC |  |  |
|  | **P** | qPCR APX LiLb_FW | CTGATGATGCTTCCCAGTGA | 62 | 95 |
|  | **Q** | qPCR APX LiLb_RV | TTGTTGAAGCGGTCGTTGTC |  |  |
|  | **R** | rt_DNApol.fw | CGAGGGCAAGACATAC | 62 | 69 |
|  | **S** | rt_DNApol.rv | GAGAGCGGGCACCAATCAC |  |  |
