# Supplementary figures and images for: Ascorbate peroxidase modulation confirms key role in Leishmania infantum oxidative defence
Source: Parasit Vectors. 2024 Nov 18;17:472. doi: 10.1186/s13071-024-06562-5 (PMC11575162; doi:10.1186/s13071-024-06562-5)

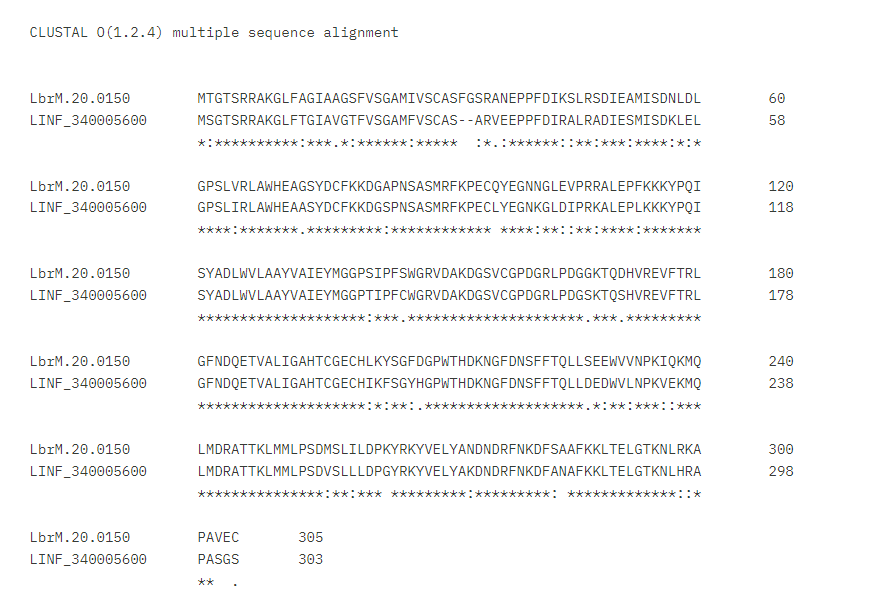

Supplement: Supplementary file 3 — Additional file 3: Figure S2. CLUSTAL O (1.2.4) multiple sequence alignment comparing the protein sequences of LINF_340005600 and LbrM.20.0150. [file 13071_2024_6562_MOESM3_ESM.png]

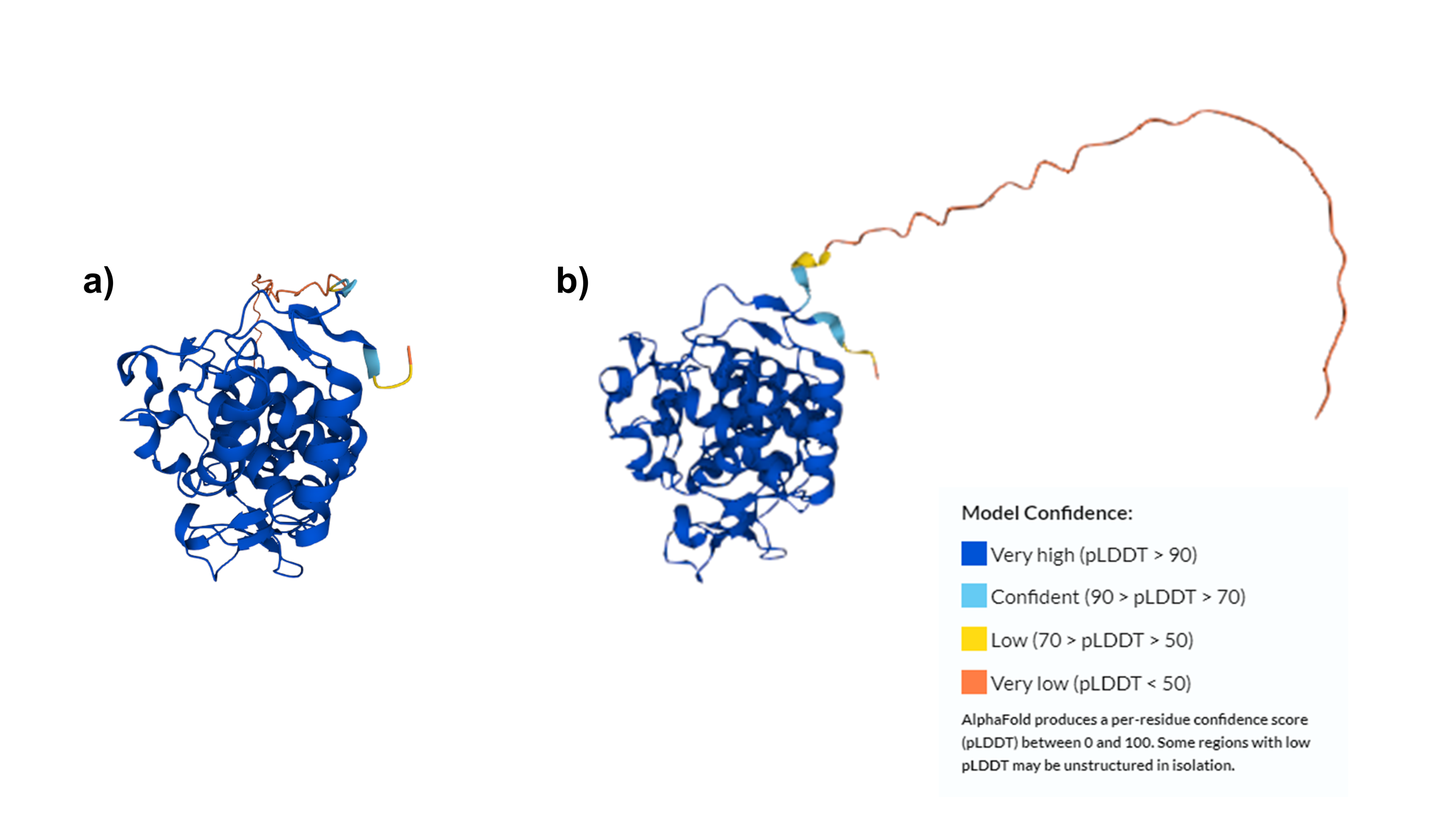

Supplement: Supplementary file 4 — Additional file 4: Figure S3. The structural model of the protein predicted by AlphaFold, where a) LINF_340005600 and b) LbrM.20.0150. The color-coded regions represent the per-residue confidence scores (pLDDT) predicted by AlphaFold. The pLDDT scores range from 0 to 100, indicating the model’s confidence in the predicted structure of each residue. Some regions with low pLDDT may be unstructured in isolation. [file 13071_2024_6562_MOESM4_ESM.png]

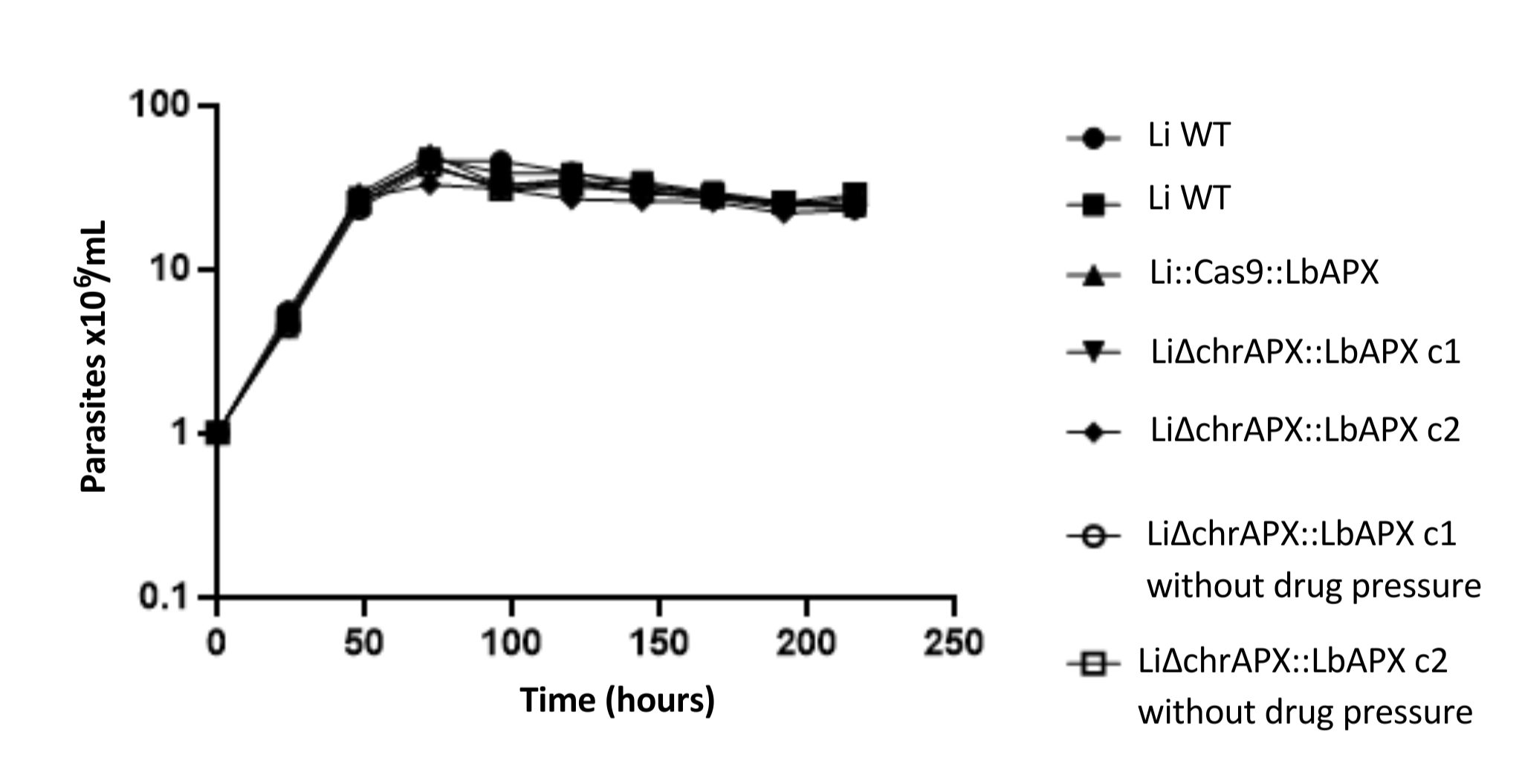

Supplement: Supplementary file 5 — Additional file 5: Figure S4. Growth of LiWT and mutant parasites. Initially, 1×10^6^ parasites per mL were inoculated in M-199 medium. The parasites were cultivated, and the growth was evaluated by daily counting the parasites using the Z1 Coulter Counter. The data presents the average of three independent experiments performed in triplicate, and the growth curves were built using a nonlinear regression model with the “beta growth then decay” equation in GraphPad Prism 9.0. [file 13071_2024_6562_MOESM5_ESM.tif]
